# Spatial synchronization of river floods growing beyond the basin boundaries in Peninsular India

**DOI:** 10.1038/s41598-025-02922-y

**Published:** 2025-05-25

**Authors:** Kanneganti Bhargav Kumar, Shailza Sharma, Rajarshi Das Bhowmik, P. P. Mujumdar

**Affiliations:** 1 Department of Civil Engineering, Indian Institute of Science, Bangalore, India; 2 Interdisciplinary Centre for Water Research (ICWaR), Indian Institute of Science, Bangalore, India

**Keywords:** Hydrology, Natural hazards

## Abstract

Floods often affect large areas and cause serious societal consequences. Despite their importance to society, the simultaneous occurrence of floods in multiple catchments is poorly understood in Peninsular India. This study aims to map regional variations in the spatial synchronization of river floods, assess changes in regional flood magnitudes, and identify regions with similar flood behaviour. The results indicate that regional flood trends exhibit a significant decline in flood magnitudes. However, the spatial dependence of floods varies regionally and has been increasing in recent times, often extending beyond the individual river basin boundaries. Five flood similarity clusters where the catchments within a cluster are likely to experience floods in closer time intervals are identified using the combination of F-madogram and Partitioning Around Medoids (PAM) clustering algorithm. Clustering results provide a basis for defining regional hydrological modeling boundaries. The synchrony of inter-basin flooding is often overlooked as the risks are measured at individual basins. The results of the study highlight strong connections of floods across space beyond the individual river basin boundaries in Peninsular India. We conclude that the regional and temporal variations in spatial flood dependencies must be considered for accurate regional flood hazard risk assessment.

**Supplementary Information:**

The online version contains supplementary material available at 10.1038/s41598-025-02922-y.

## Introduction

Simultaneous flooding of multiple rivers has become increasingly common worldwide in recent years^1–5^. When one river floods, nearby rivers often experience flooding at the same time. Large-scale flood events spanning vast regions have been documented with increasing frequency^4,6–12^. India is particularly vulnerable to this phenomenon due to the large-scale monsoon systems and their interactions with landscape conditions. Numerous studies have highlighted the occurrence of extensive flooding across multiple Indian river basins^7,13–18^. For instance, Parthasarathy et al.^7^ analysed the areal extents of summer monsoon floods over a period of 114 years (1874–1984) in India. Authors identified 1961, 1933, 1892, and 1983 as the worst flood years which affected 44.5%, 43.2%, 42.5% and 42.1% of India’s total area, respectively. Roxy et al.^19^ also analysed several historical floods that caused significant loss of human lives and property, coinciding with some of the most intense and widespread extreme storm events. Examples include the Central India floods of 1989 and 2000, and the South Asian floods of 2007. A recent study by Nanditha and Mishra^20^ also highlighted an increasing probability of widespread flooding in seven major river basins of Peninsular India. These widespread and concurrent flood events pose significant challenges for disaster management and the (re)insurance industry, magnifying the financial risks. Hence, understanding the synchronization of floods across multiple catchments can provide insights for regional flood mitigation planning and management.

Previous research has primarily focused on flood events within individual basins or regions, often examining a limited number of events or areas^4,10,11,17,20,21^. Studies that quantify the spatial extent of floods across multiple basins are scarce^1,22^. Understanding the spatial dependencies between flood events is crucial for the accurate modeling of regional flood risks. While various models capture different types of dependencies, selecting the most appropriate approach requires knowledge of spatial dependence within a region. To address this gap, Berghuijs et al.^1^ introduced the concept of the “flood synchrony scale” to measure the spatial scale of cross-basin floods, while Brunner et al.^23^ and Brunner and Fischer^24^ used “flood connectedness” to examine the spatial dependence of flood events across distant regions, utilizing complex network theory. By employing complex network theory, we can map pairs of catchments with similar streamflow synchronization, which might not necessarily lie on the same river network. This approach even provides flexibility to map distant pairs of catchments, whereas the flood synchrony scale, which calculates the distance for which at least 50% of neighbouring stations experience floods simultaneously, is limited in its applicability in this context. In most of the river basins in India, the expected time window of flood occurrence is July last week to mid-August due to the Indian summer monsoon^25^. Hence, there is a high chance of synchronization of floods, even in distant catchments of Peninsular India during this period.

Anthropogenic climate change leads to regional changes in water availability^26^ and the risk of floods^27^. The trends in river flood magnitudes across 70 individual catchments in Peninsular India are identified by Sharma and Mujumdar^28^. However, a comprehensive analysis of regional flood trends across all catchments will explicitly describe the large-scale variation of spatial patterns of floods^29^. In addition, this will identify the hotspots of changing regional flood magnitudes in the warming climate. The spatial dimension of floods is often neglected in risk assessment of high-impact floods^22^. Regional flood risk assessments contravene the fundamental principles and usually focus on individual locations^30^. The clustering of regions where floods show similar behaviour might help understand the nature of spatially extensive floods. Identification of flood similarity clusters will provide a strong foundation for regional flood hazard and risk assessments in Peninsular India.

The aim of this study is threefold: (1) to assess the connectedness measure that enables the mapping of regional differences in spatial flood dependence across different time periods, (2) to estimate regional trends of annual maximum floods, and (3) to identify the flood similarity clusters and analyze attributes of catchments falling within the clusters in Peninsular India. The following sections outline the study area and data, the methodological approach, and the metrics used. We examined a window of 1–5 days for coincident peak flow annual maxima across 137 catchments during the period 1980–2018. We then mapped regional differences in spatial flood dependence in the past (1980–1999) and recent period (2000–2018), calculated the changes in connectedness at each catchment, analyzed decadal regional flood trends, and identified flood similarity clusters using the combination of F-madogram^31^ and Partitioning Around Medoids (PAM) clustering algorithm^32^. The results from this study improve our understanding of the spatial dependence of floods, which is crucial for improving large-scale flood simulation models and understanding how future widespread flood risk may change in Peninsular India.

## Study area and data

Peninsular India is a large region with a gradient of increasing continental climate from south to north and a gradient of growing elevation from east to west. These gradients influence precipitation and temperature patterns, which have implications for streamflow regimes and flood generation. Moisture fluxes are driven by the prevailing southwest monsoon, resulting in high-intensity precipitation events often associated with cyclonic weather patterns. During this season, the surface pressure gradient directs south-westerly (SW) monsoon winds in the lower troposphere from the Indian Ocean toward land. Peninsular India receives a major portion of its annual total rainfall during the SW monsoon season (June to September). The northeast monsoon (October to December), also known as the winter monsoon, is another major and prominent feature of the Indian subcontinental climate system. Warm and moist air from the Bay of Bengal moves from northeast to southwest during this season, occasionally leading to intense precipitation events and severe flooding, particularly over the eastern and southeastern regions of Peninsular India.

**Fig. 1:**
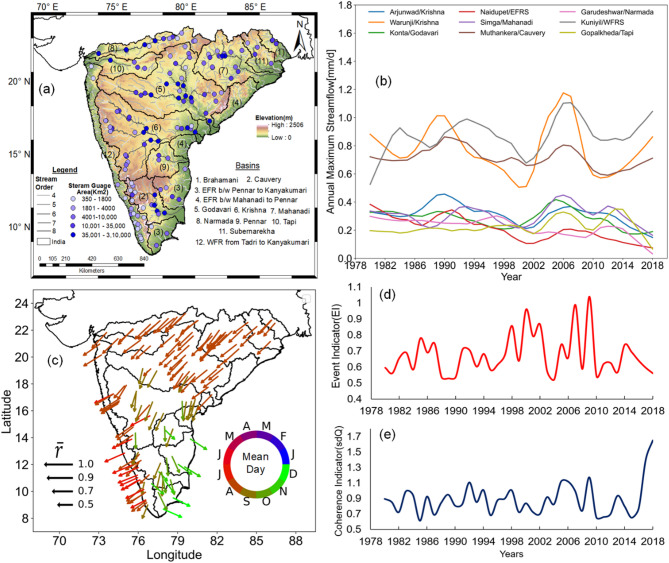
(**a**) Study area map showing elevation, river basins, stream network, and gauge locations with the size of the catchments represented by varying colors of points, (**b**) smoothed annual maximum (AM) flood series for selected stations, highlighting synchronized flood patterns across varied environments, (**c**) flood seasonality for 137 gauges, visualized by arrows indicating average flood timing and concentration and (**d**,**e**) time series of event-type indicator (EI) and coherence indicator (sdQ). EI shows no significant trend, indicating consistent patterns in flood distribution over time. The map is prepared in QGIS (Version 3.34.11-Prizren (2024), URL: http://qgis.org), and other parts of the figure are generated using R (Version 4.4.2. (2022), URL: https://www.r-project.org).

A total of 137 gauging stations with daily discharge data spanning from 1980 to 2018 are selected from CAMELS-IND dataset for this study^33^. The spatial distribution of these stations across 12 river basins, along with their corresponding elevation and stream order, is presented in Fig. 1a. The gauge selection criterion emphasized comprehensive coverage of the study region to capture diverse flood-generating mechanisms. Stations with missing data exceeding 20% within the specified period were excluded. Additionally, catchments smaller than 100 km^2^ are omitted due to the potential influence of land-use changes and flood retention on hydrological processes in these areas.

Floods are shaped by the interplay of processes in the atmosphere, catchments, and river networks. The spatial coherence, i.e., the synchronous occurrence of floods in the spatial domain, is the consequence of the similarity in one or several of these processes^11^. Figure 1b of the smoothed time series of annual maxima shows the synchronous behavior in flood magnitude in nine randomly selected stream gauges. The stations cover high mountainous range west flowing rivers with smaller to medium catchment areas (Gopalkheda/Tapi, Kuniyil/WFRS and Garudeshwar/Narmada) and medium mountainous range east flowing rivers with medium to large catchment areas (Konta/Godavari, Arjunwad/Krishna and others). Even though these stations are in different basins with a possible broad spectrum of climate, landscape characteristics, and flood-generating mechanisms, the synchronization among the flood series is remarkable. The flood series show parallel patterns over time, with periods of high and low flood activity occurring simultaneously across multiple basins.

The mean day of flood occurrences and strength of occurrences (represented by the persistence, $\overline{r}$) are calculated with circular statistics^34^ based on the dates of floods. The $\overline{r}$ value ranges from 0 to 1, where 1 indicates that annual maxima floods occur on the same day of the year, while lower values indicate a wider spread in timings of annual maxima floods. The spatial pattern of the mean within-year flood timing between 1980 and 2018, as shown in Fig. 1c, provides further support for emphasizing the synchronization in flood series across Peninsular India. The mean timings of floods at many catchments are July with strong persistence (*r* values are higher, Fig. 1c), except for a few catchments in the delta regions of the Cauvery and Krishna basins. These delta regions receive more rainfall during the northeast monsoon period.

The variability of flood occurrences, *r*, is shown in Fig. 1c. However, this measure cannot effectively distinguish between scenarios where floods are spread throughout the year and those where floods are concentrated in a few events, with these events occurring at opposite times in a directional space. Therefore, in this study, an Event Indicator (EI)^11^ is used to quantify the event-type strength of a certain hydrological year. This indicator should determine whether annual maximum streamflow (AMS) values across Peninsular India are concentrated in a few events (event-type) or are spread throughout the year (non-event-type). The AMS values in Peninsular India show event type with no significant increase in event type strength over time (Fig. 1d). It indicates that flood timings tend to spread out less in Peninsular India, which further emphasizes the occurrence of large-scale flooding across the basins. The magnitude of coherence here is the standard deviation of discharge anomalies at all the basins, showing that the AMS values at multiple basins will have high and low periods simultaneously (Fig. 1e). It is evident that floods in Peninsular India show synchronization across multiple basins. Hence, it is necessary to investigate the spatial dependence of floods at multiple basins in the study area.

## Methods

### Co-occurrence of floods and complex networks

This study investigates the co-occurrence or synchronization of pairwise annual maxima. For each pair of stations and each year, annual maxima are considered to co-occur if they occur on the same day, with a tolerance of ± 5 days. Figure S1 shows the variation in sum of pairs of catchments whose co-occurrence frequency is greater than the threshold with varying window lengths in days. This tolerance is considered to account for travel times, which refers to the phenomenon where event occurrences might be slightly time-shifted between upstream and downstream locations^1,35–37^. The frequency of co-occurrence (or synchronization) of annual maxima discharge between stations $S_{i}$ and $S_{j}$ is denoted as $F_{Co}$ and computed using

1 $$F_{co} \left( {S_{i} ,S_{j} } \right) = \frac{{\# \left| {Pairs\;S_{i} \& S_{j} \; has\;difference} \right| \le 5 \,days}}{N }$$

where *N* is the number of joint data points in the two stations $S_{i}$ and $S_{j}$. A higher synchrony value indicates a greater percentage of extreme floods occurring on the same day (within ± lag time). When analyzing co-occurrence between multiple pairs of time series, especially in small sample sizes, traditional significance tests may become unreliable due to the increased chance of Type-I errors, where random patterns are incorrectly identified as significant^37,38^. To address this, the present study used a combination of false discovery rate (FDR) correction and bootstrap resampling. The co-occurrence matrix is computed for all pairs of stations, and significant co-occurrence frequencies (α = 0.05) are extracted. FDR correction, as proposed by Benjamini and Yekutieli^38^, is applied to control the overall rate of false positives across multiple hypothesis tests. The procedure involves ordering all the *p-*values from the tests (m) in increasing order. Let *α* be the significance level, here $\alpha = 0.05$, and $p_{\left( i \right)}$ denotes the *i*th ordered *p* value. Let *k* be the largest index value of *i* for which it satisfies the constraint

2 $$p_{\left( i \right)} \le \frac{i}{m}\alpha$$

Then $p_{\left( k \right)}$ will be the *p*-value threshold used for rejecting the independence hypothesis. For the first *k p*-values, the null hypothesis is rejected, indicating significant co-occurrence.

Using the significant pairwise dependence measure, we map the complex network of floods using the R-package *igraph*^39^ to evaluate the strength of spatial connectedness of floods. This approach enables the visualization and description of structures and connections within large datasets^35,40–42^. The weighted networks are constructed by using Kendall’s correlation as edge weights to assess the strength of the relationship between pairs of catchments. Edges with correlation below a specified threshold of 0.5 are removed, and this threshold is chosen to include substantial correlations without excluding too many potential relationships. A binary matrix is then created, highlighting the flood events that impacted different catchments. Subsequently, a network of flood co-occurrences is generated using the binary matrix of total flood occurrences, and the flood connectedness of a station with other stations is quantified. Flood connectedness is defined as the number of catchments that co-experience flood events with a given catchment. The binary information is stored in Adjacency matrix A, which is used to build the complex network graph $G = \left( {V,E} \right)$, where *V* represents vertices, i.e., network nodes (in our case catchments), and *E* represents edges, i.e., links connecting the nodes (in our case connecting pairs of catchments co-experiencing flood events). The measure of connectedness and the measure of connectedness length for each catchment are computed using the above network. Connectedness is described by the network degree (also called centrality degree), which defines the number of edges incident on a particular node, indicating how many catchments are connected with a specific catchment. Connectedness length is defined for each existing edge as the Euclidean distance between catchment outlets of concurrent floods but not necessarily physically connected catchments. The connectedness is estimated for the whole period (1980–2018), the past period (1980–1999), and the recent period (2000–2018). In addition, a relative change in degree is also estimated to understand the changes in spatial synchronization of floods with time.

### Estimation of regional trends

Trends in annual maximum flood discharge are detected using the Mann–Kendall trend test^43^, and the slope of linear trends is estimated using the Theil-Sen slope estimator^44^. Trends are expressed in units of percentage per decade to facilitate comparison across catchments of varying sizes^12,45–47^, such that

3 $$T = \frac{S \times 10\,years}{{\overline{x}}} \times 100$$

where *T* is the trend in %/decade, *S* is the Sen’s slope and $\overline{x}$ is the mean of the annual maximum discharge time series. Regional spatial patterns are generated using the autoKrige function of the R automap package^48^ by interpolating the decadal flood trends across space. The function is based on automatic variogram fitting to preserve the spatial structure of original data and predicts values at unobserved locations using data from observed locations. The uncertainty of the regional flood trends is estimated using the block kriging standard deviation as it has the advantage of lowering prediction errors.

### Clustering algorithm for extremes

Clustering algorithms are widely used to study spatial or temporal patterns in hydrology and climate sciences. However, popular algorithms like the *k*-means algorithm identify clusters based on minimizing the variance within each cluster. This concept makes them ideal for applications concerned with finding patterns with respect to mean behaviors; whereas their suitability in the context of extremes is questionable. Bernard et al.^49^ developed a clustering procedure for maxima by combining the Partitioning Around Medoids (PAM) clustering algorithm^32^ with a measure of pairwise dependence *F*-madogram^31^ explicitly suitable for extremes to overcome the limitations of variance-based clustering approaches. This combination provides an efficient, nonparametric, and theoretically sound clustering algorithm for extreme values^50^.

PAM algorithm uses representative objects called medoids to represent each cluster without averaging the original maxima values and, therefore, preserves the max-stable property of maxima. *F*-madogram provides a dimensionless metric that compares the shape of extreme value distributions between two stations. The random variables $M_{i}$ and $M_{j}$ represent maxima of daily discharge data at stations *i* and *j*, respectively. Given the samples of bivariate maxima $\left( {M_{i}^{\left( t \right)} ,M_{j}^{\left( t \right)} } \right)^{T}$ at *T* time units, the nonparametric *F*-madogram estimator is defined as

4 $$\hat{d}_{ij} = \frac{1}{2T}\mathop \sum \limits_{t = 1}^{T} \left| {\hat{F}_{i} \left( {M_{i}^{\left( t \right)} } \right) - \hat{F}_{j} \left( {M_{j}^{\left( t \right)} } \right)} \right|$$

where *T* is the bivariate sample length and $\hat{F}_{i}$ is the empirical distribution function

5 $$\hat{F}_{i} \left( u \right) = \frac{1}{T}\mathop \sum \limits_{t = 1}^{T} 1_{{\left\{ {M_{i}^{\left( t \right)} \le u} \right\}}}$$

where $1_{{\left\{ {M_{i}^{\left( t \right)} \le u} \right\}}}$ is the indicator function for the event $\left\{ {M_{i}^{\left( t \right)} \le u} \right\}.$ This clustering algorithm is based only on the shape of extreme value distribution and not on the magnitude of extreme events or geographic proximity, which can result in misclassification. Bracken et al.^50^ suggested a modification to this algorithm to incorporate the physical proximity of the stations. The modification to *F*-madogram is

6 $$\widehat{{\hat{d}}}_{ij} = \hat{d}_{ij} + p_{ij}$$

where $p_{ij} = \frac{{q_{ij} }}{{\mathop \sum \nolimits_{i = 1}^{N} q_{ij} }}{{\max}}\left( {\hat{d}_{ij} } \right)$ and $q_{ij} = \sqrt {\left( {x_{i} - x_{j} } \right)^{2} + \left( {y_{i} - y_{j} } \right)^{2} }$. A scaling factor based on the Euclidean distance $q_{ij}$ between the stations is added such that the largest value of *F*-madogram will never exceed. This modification significantly improved the cluster coherence^50^. The relevant number of clusters is selected using the silhouette coefficient^51^, which compares cluster tightness with cluster separation.

## Results

### Connectedness of annual maxima floods

**Fig. 2:**
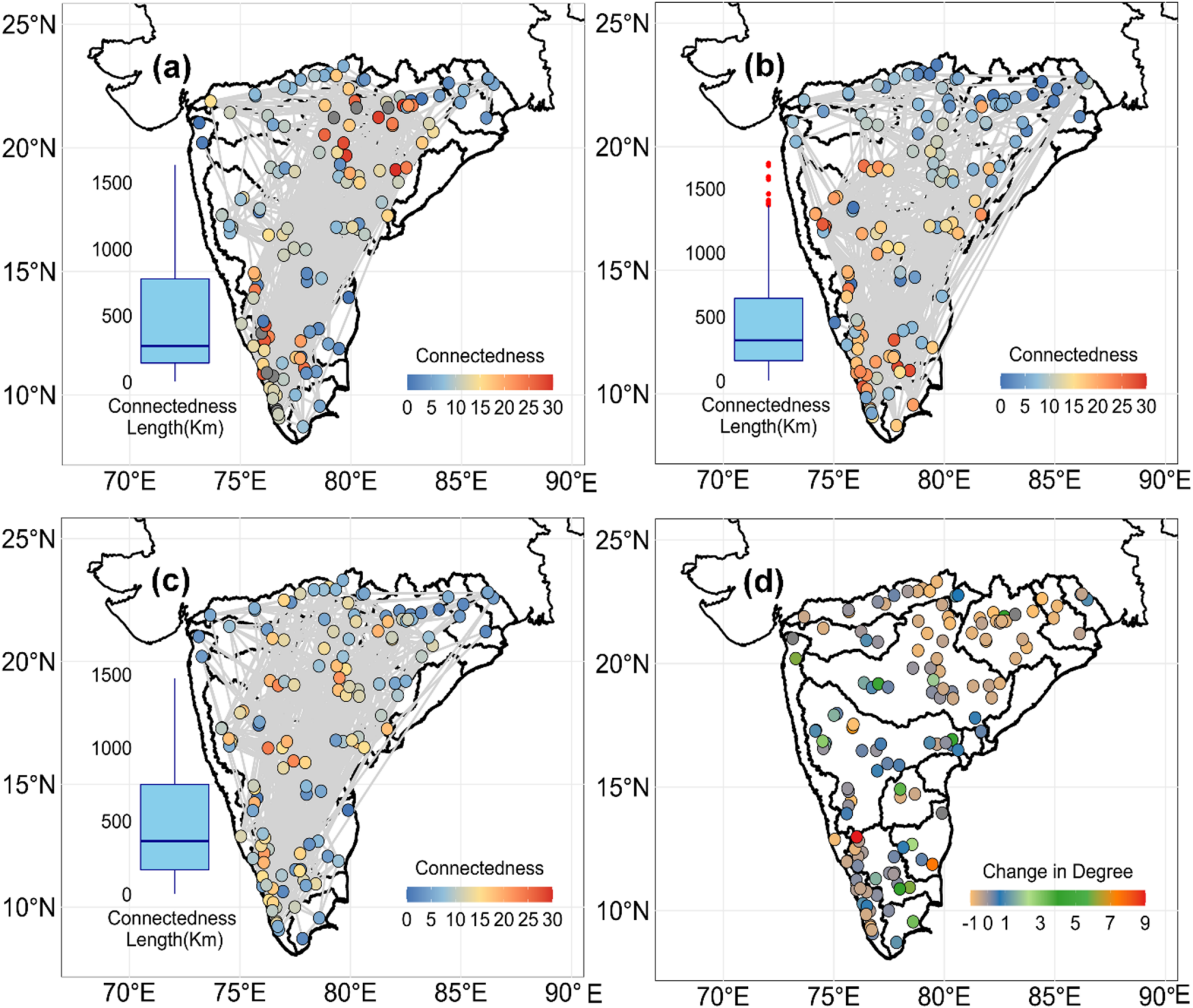
Co-occurrence networks of floods in Peninsular India. Connections (edges) are plotted between pairs of catchments, and vertices are colored by connectedness (degree). Comparison of networks: (**a**) past (1980–1999) and (**b**) recent (2000–2018) periods, (**c**) overall network of floods for the period of 1980–2018. An increase in connectedness has been observed in recent years, and the relative change in the difference in connectedness is shown in (**d**). The increase in connectedness indicates that catchments in Peninsular India are likely to experience higher simultaneous floods in Peninsular India. The figure is prepared in R (Version 4.2.2 (2022), URL: https://www.R-project.org/).

The connectedness and connectedness length of pairwise annual maxima floods are presented in Fig. 2. The results show that flood connectedness varies across both space and time. The co-occurrence networks generated using 3-day and 7-day window lengths also show flood connectedness across the space (Fig. S2). A significant increase in connectedness is observed during the recent period (2000–2018) (Fig. 2b) compared to the earlier period (1980–1999) (Fig. 2a). During both periods, floods co-occurred across basins, with a median connectedness length of 180 km and 200 km in the earlier and recent periods, respectively. The maximum connectedness length was 1500 km in the earlier period and 1600 km in the recent period. The overall flood network (Fig. 2c) highlights high connectedness across multiple basins, with the highest values observed in the Krishna and Godavari basins. The median connectedness length is 350 km for floods of the whole period, with a maximum length of 1450 km. Figure 2d shows the relative change in connectedness, with an overall increase in connectedness in annual maxima floods. An increase in flood connectedness over time may be attributed to changes in the spatial dependencies of flood drivers such as rainfall, soil moisture, or anthropogenic factors. To explore this further, we examined the connectedness in rainfall (RF) and soil moisture (SM) extremes as shown in Fig. S3. Both variables exhibited higher connectedness across basins compared to flood connectedness at most nodes. Connectedness in rainfall and soil moisture extremes has also increased over time. However, addressing how this increased connectedness of drivers influences flood connectedness is beyond the scope of this study.

The catchments that are not physically connected have shown flood co-occurrence over long distances. For instance, the catchments in the Subarnarekha and Brahmani basins are co-experiencing the floods with catchments in east-flowing rivers between the Pennar and Kanyakumari basins. Similarly, the floods in the Narmada and Mahi basins are synchronized (co-occurrence) with catchments in Cauvery and west-flowing rivers. To further illustrate this, a regional flood event of the year 1989 that affected a large number of catchments simultaneously is shown in Fig. S4. In 1989, a total of eight spatial flood events were identified, with events defined by concurrent flooding across the catchments within a tolerance of ± 5 days. In Event 4, approximately 106 catchments experienced floods concurrently, with a mean day of occurrence on the 205th day of the year and a mean streamflow of 0.360 mm (± 0.352 mm). The next major event, Event 5, involved around 18 catchments with a mean streamflow of 0.22 mm (± 0.47 mm). The event indicator (EI), shown in Fig. 1d, further highlights the strength of regional flood occurrences in Peninsular India. These long-distance connections indicate a consistent pattern of flood co-occurrence across multiple basins. However, some catchments in the Mahanadi, Narmada, and Wainganga basins show a decrease in connectedness, with a maximum relative change of − 0.85 with respect to the recent period. In contrast, many catchments show increasing connectedness, indicating a higher likelihood of widespread flooding.

### Regional flood trends

**Fig. 3:**
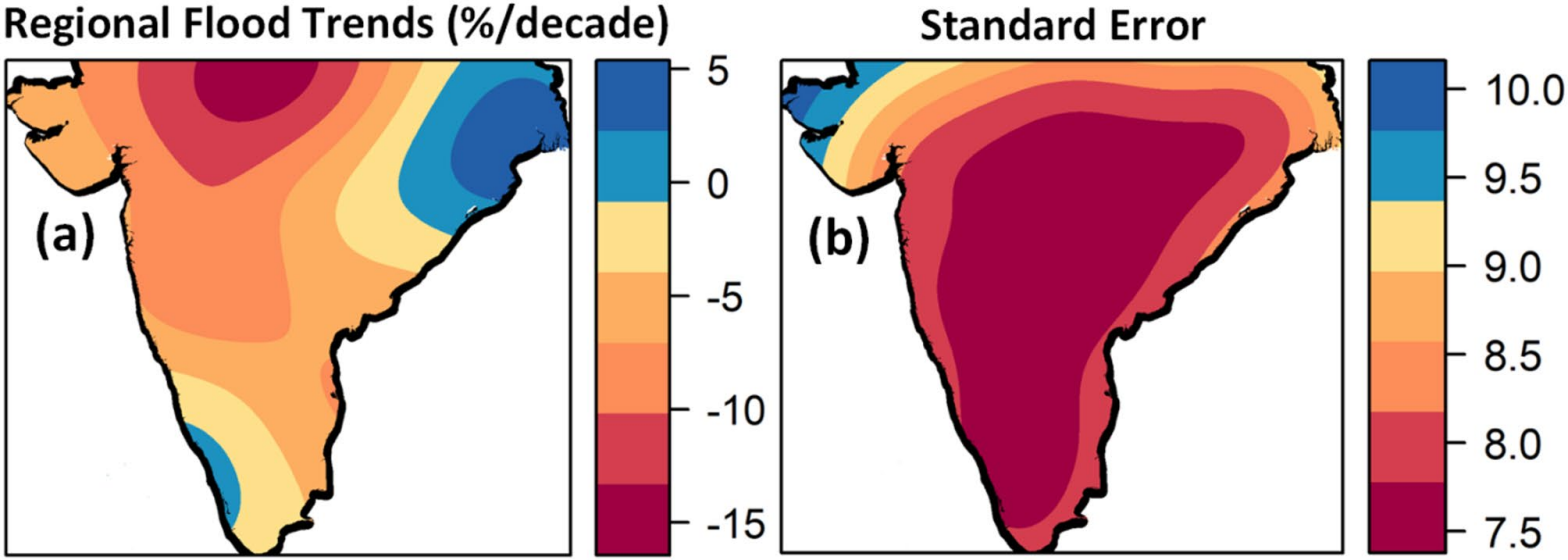
(**a**) Regional trends of annual maximum river floods in Peninsular India for the period 1980–2018 and (**b**) uncertainties of regional trends in terms of standard deviation estimated at a block size of 100 × 100 km^2^. The maps are generated in R (Version 4.2.2 (2022), URL: https://www.R-project.org/).

Regional patterns of increases and decreases in annual maximum flood magnitudes for 39 years are shown in Fig. 3. A dominance of declining trends in river flood magnitudes with a substantial decline in the Narmada River basin is observed in Peninsular India. A slight increase in the flood trends is observed in the west-flowing rivers from Tadri to Kanyakumari. Increasing trends in floods are more pronounced in Subernarekha, Brahmani, and lower Mahanadi River basins. Regional flood trends range between an increase of + 5% to a decrease of − 15.1% per decade relative to the mean flood discharge (Fig. 3a). The uncertainties of the regional trends vary between 7.55 and 10% per decade (Fig. 3b). Local trends at streamflow gauges range between a maximum of + 12.65% and a minimum of − 26.77% (Fig. S5). Out of 137 streamflow gauging stations, 45 stations (~ 32.8% of stations) show increasing trends in river floods. These trends are estimates of long-term changes in annual maximum flood discharge, which have implications for the design of flood protective measures and planning preventive actions against high-risk floods.

### Flood similarity clusters

**Fig. 4:**
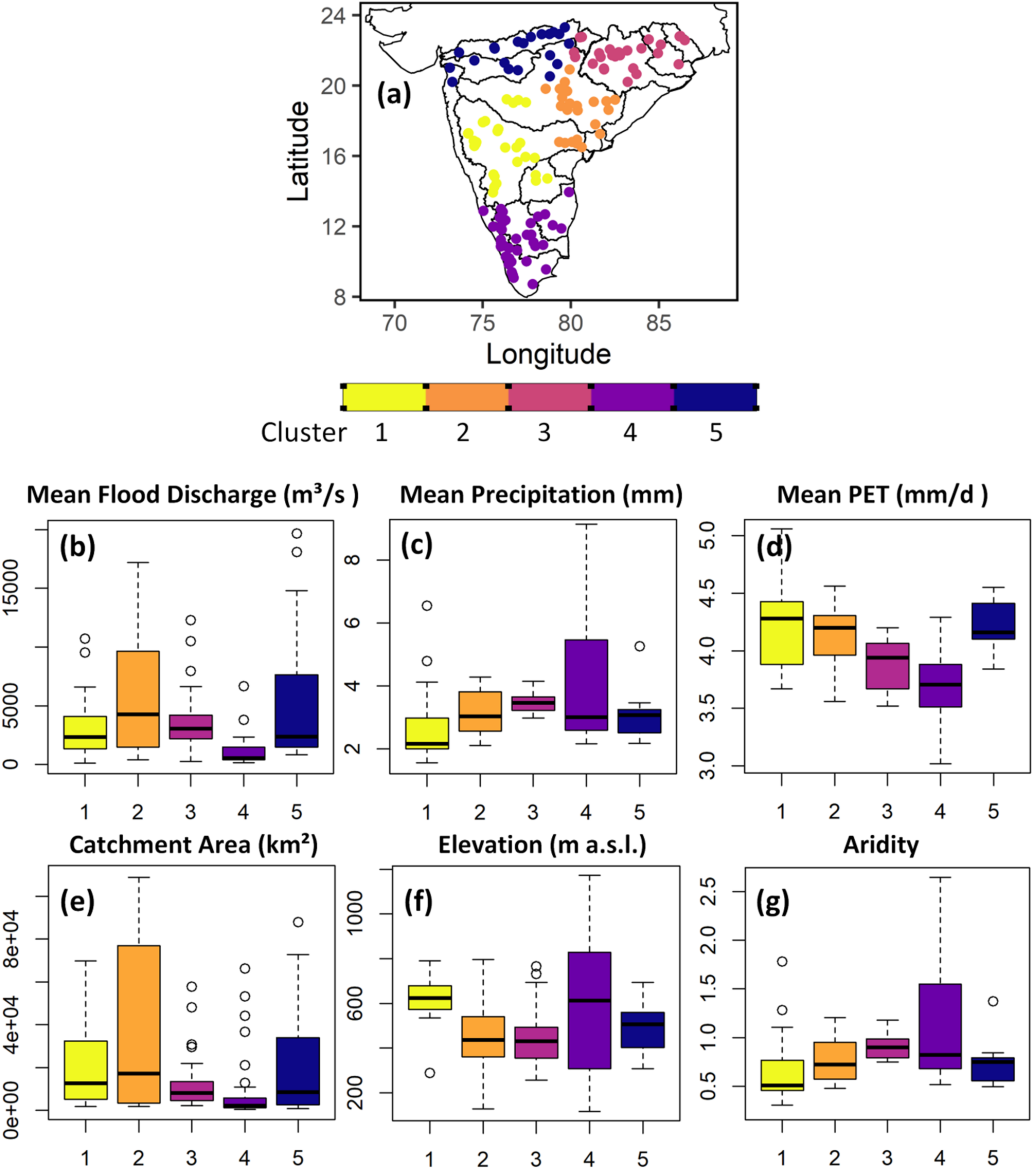
(**a**) Five flood similarity clusters derived using an F-madogram based extreme value clustering algorithm and (**b**) catchment attributes of the clusters. The figure is prepared in R (Version 4.2.2 (2022), URL: https://www.R-project.org/).

Five flood similarity clusters are identified using the annual maximum flood discharge as input to the F-madogram based Partitioning Around Medoids (PAM) clustering algorithm (Fig. 4a). Catchment attributes of the clusters are also presented to provide a detailed description of topographical and climatological characteristics (Fig. 4b–g). Cluster 1 includes the catchments of the Godavari middle subbasin, Krishna basin except the lower subbasin and Pennar river basins. The subbasin details of the Godavari and Krishna river basins are shown in Figs. S6 and S7, respectively. The catchments of cluster 1 have the lowest daily mean precipitation and higher potential evapotranspiration. These catchments have lower aridity index values (P/PET), and according to the United Nations Environmental Programme (UNEP) classification, these catchments have semi-arid (AI 0.2–0.5) to dry sub-humid climate (AI 0.5–0.65) climate conditions. These catchments have a higher elevation compared to other clusters. These catchments generally receive maximum rainfall during the southwest monsoon. Annual maximum floods occurred in most of the catchments of cluster 1 during September and occurred in August in a few catchments.

Cluster 2 includes catchments of lower Godavari, Indravati, lower Weinganga, and lower Krishna sub-basins. Mean flood discharges are higher in cluster 2 compared to other clusters and the catchment areas are also large. The climate of these catchments varies between sub-humid and humid. The mean elevation of cluster 2 and cluster 3 is almost the same. Annual maximum floods in both clusters occurred in August. Cluster 3 includes catchments of the Mahanadi basin, Subernarekha basin, Brahmani and Baitarni river basins, lower Narmada, and a few catchments of Weinganga subbasin. The mean flood discharges are lower than cluster 2 but higher than other clusters. The climate conditions of cluster 3 catchments are humid. Cluster 4 includes catchments of the Cauvery River basin, west-flowing rivers between Tadri and Kanyakumari, and east-flowing rivers between Pennar and Kanyakumari. Mean flood discharges are lowest in cluster 4 and the catchments have a humid climate. The maximum mean precipitation is highest in these catchments and the mean elevation is also very high. Mean potential evapotranspiration is lowest and the catchment sizes are the smallest among all the clusters. Cluster 4 shows the occurrence of annual maximum floods during the southwest monsoon in west-flowing rivers between Tadri and Kanyakumari and floods occurred during the north-east monsoon in Cauvery and east-flowing rivers between Pennar and Kanyakumari. Cluster 5 includes all the catchments of the Narmada and Tapi river basins, west flowing rivers between Tapi and Tadri and a few catchments of the Weinganga and Wardha subbasins. The climate conditions in these catchments are dry, sub-humid, and humid. Most of the annual maximum floods occurred in August month in cluster 5.

## Discussion

The simultaneous occurrence of floods across multiple basins presents a critical challenge for mitigation strategies, often resulting in widespread flooding. Large-scale meteorological circulation systems can influence both the occurrence and co-occurrence of extreme events like floods^1,10,22^. This study observed a high synchronization of annual maxima floods, corresponding to an increase in spatial connectedness. This suggests that the spatial extent of flood co-occurrence is not limited to adjacent or physically connected basins but can extend across widely separated regions, influenced by similar meteorological processes and surface conditions^9,37^. Such results are crucial, as they indicate that flood risks are interconnected across the basins, necessitating inter-basin cooperation in flood management. Recent studies have also documented similar trends, with increasing flood synchronization observed in regions such as Europe^1,5^ and China^4^. These studies emphasize that the simultaneous occurrence of floods is becoming more frequent and is expected to intensify due to climate change. This growing synchronization underscores the need for enhanced forecasting, preparedness, and coordinated flood mitigation efforts across large spatial scales.

Changes in river flood magnitudes have implications for the design of flood control structures. Increasing trends in extreme rainfall are reported in India^19,52^. However, observed increases in rainfall extremes do not appear to have translated to river floods in many regions across the globe^53^. Ivancic and Shaw^54^ studied 390 watersheds across the contiguous US and found that only 36% of 99th-percentile extreme rainfall events lead to a corresponding 99th-percentile extreme discharge. Global studies provide more evidence of significantly decreasing flood trends than increasing trends^55^. We observe a dominant regional pattern of decreasing flood trends in Peninsular India with increasing flood trends in a few catchments. Therefore, it can be concluded that an increase in extreme rainfall does not necessarily lead to an increase in flood magnitudes in Peninsular India.

Most of the flood-related studies are limited to the individual river basins in India^56,57^. Nanditha and Mishra^20^ observed an increase in widespread floods in India; however, the spatial analysis is performed within individual river basins. Quantification of the risk of floods, which simultaneously affect many rivers, needs immediate attention. This study identifies five spatial clusters where river floods show similar behaviour in Peninsular India. The results are useful for investigating the flood risk for ungauged basins located in the flood similarity regions. The catchments within a cluster are more likely to experience floods in closer time succession and therefore the information on flood similarity clusters is useful in planning flood management strategies and setting up the boundaries for regional flood modeling studies. As climate change exacerbates flood risks, insights from regions with similar flood characteristics might help tailor region-specific mitigation strategies, enhancing preparedness and response efforts.

## Conclusions

This study employed complex network theory to analyse the spatial connectedness and length of connectedness of annual maxima floods in Peninsular India. The results indicate that simultaneous flood events have become more frequent across multiple basins in recent times despite a significant decrease in regional flood trends. Additionally, five distinct flood similarity clusters are identified, suggesting a higher likelihood of synchronized flooding within the catchments of these clusters. These findings underscore the need for a comprehensive understanding of large-scale flood risk, particularly in regions influenced by monsoon systems. Our findings have important implications for future regional flood hazard and risk assessment to ensure long-term sustainability with the changing climate.

One potential limitation of the present study is that we performed the analysis using annual maximum floods. The study can be further extended by adopting a peaks over threshold (POT) approach that could increase the sample size and potentially enhance the strength of flood connectedness. Nevertheless, the annual maxima approach has demonstrated significant flood connectedness across basins, including long-range connections. In addition, several reservoirs operate for a variety of purposes in Peninsular India. The presence of reservoirs can modulate the spatial connectedness of floods in human-modified systems. This aspect is critical to provide a comprehensive understanding of the impact of human interventions on flood connectedness across catchments. Present study can be further extended using spatial statistical models and hydrological models to address the effect of flow regulations and climate change on the spatial synchrony of river floods.

## Electronic supplementary material

Below is the link to the electronic supplementary material.

Supplementary Material 1

## Data Availability

The streamflow data and catchment attributes used in this study are obtained from the CAMELS-IND^33^ dataset, which is freely available at 10.5281/zenodo.14005378.
