# Supplementary material for: Spatial synchronization of river floods growing beyond the basin boundaries in Peninsular India
Source: Sci Rep. 2025 May 25;15:18160. doi: 10.1038/s41598-025-02922-y (PMC12104415; doi:10.1038/s41598-025-02922-y)
Supplement: Supplementary file 1 — Supplementary Material 1 [file 41598_2025_2922_MOESM1_ESM.pdf]

Spatial synchronization of river floods growing beyond the basin boundaries in Peninsular India

Kanneganti Bhargav Kumar[1], Shailza Sharma[1*], Rajarshi Das Bhowmik[2] and P. P. Mujumdar[1,2]

[1]Department of Civil Engineering, Indian Institute of Science, Bangalore, India

[2]Interdisciplinary Centre for Water Research (ICWaR), Indian Institute of Science, Bangalore, India

Correspondence to [*]shailzas@iisc.ac.in

Content of this file: Figures S1 to S7

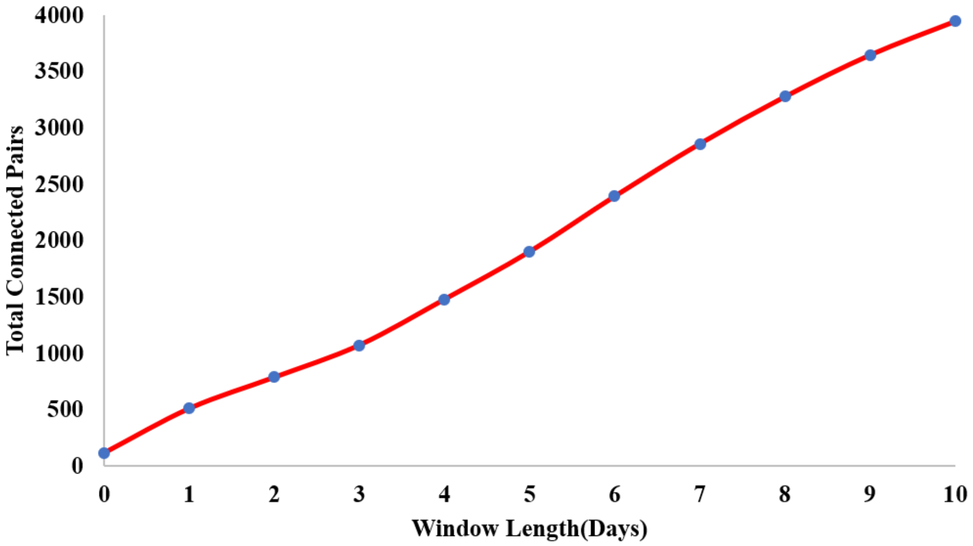

Figure S1. The variation in total connected pairs with window length (days) in Peninsular India.

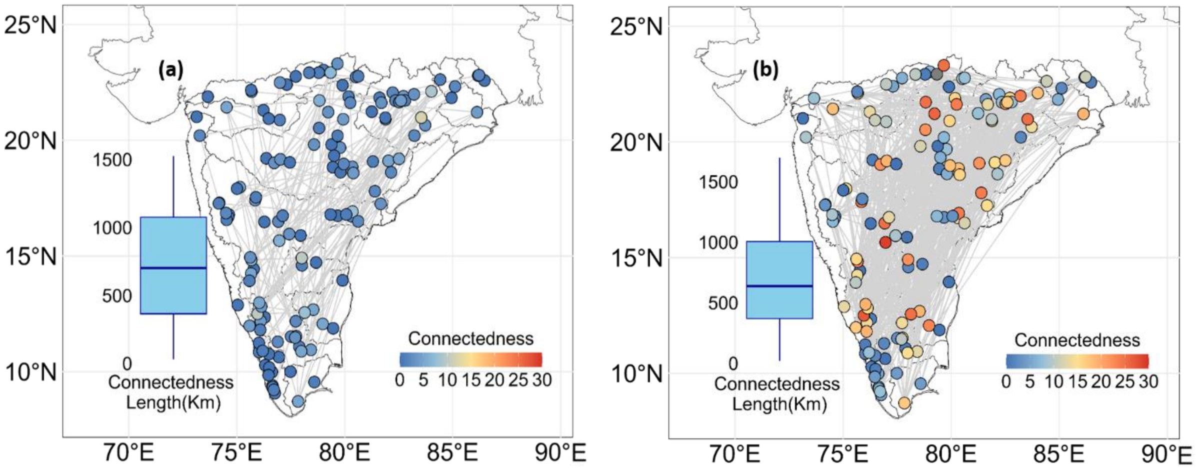

Figure S2. Co-occurrence networks of floods in peninsular India for 3-day and 7-day window lengths.

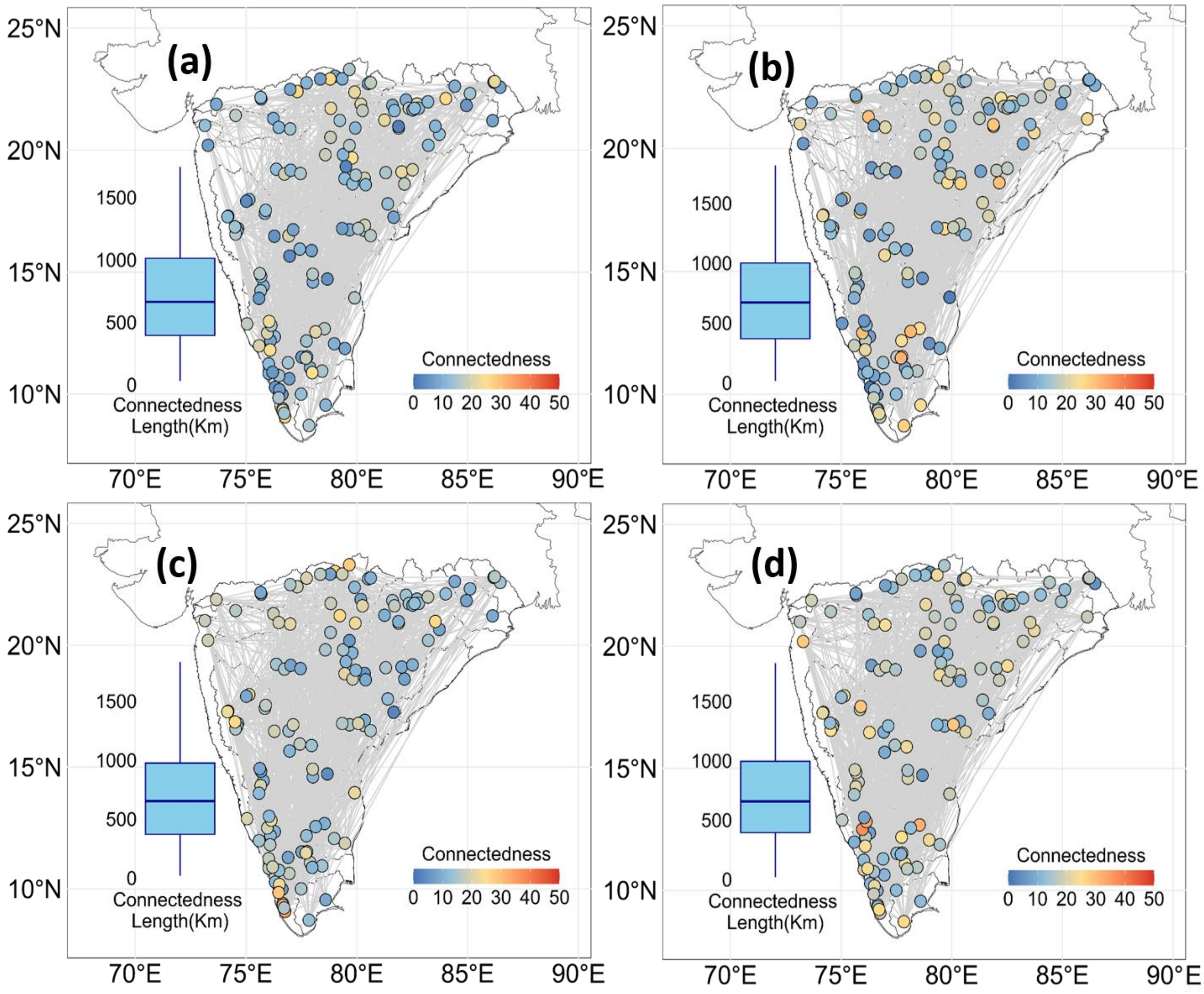

Figure S3. Co-occurrence networks of flood drivers, namely rainfall (RF) and soil moisture (SM), in Peninsular India. Connections (edges) are shown between pairs of catchments, with vertices coloured by connectedness (degree). Rainfall networks for the past (1980–1999) and recent (2000–2018) periods are shown in panels (a) and (b), respectively. Soil moisture networks for the past (1980–1999) and recent (2000–2018) periods are shown in panels (c) and (d), respectively.

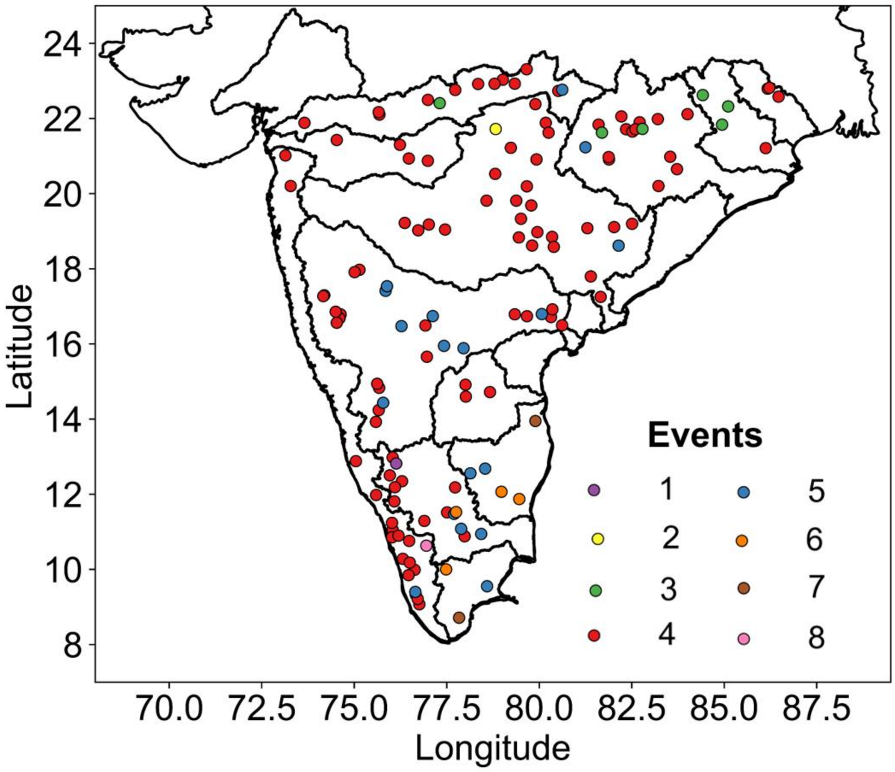

Figure S4. Regional Annual maxima floods in year 1989. The groups are made based on day of occurrence of flood event. Major catchments are experienced floods simultaneously in year 1989.

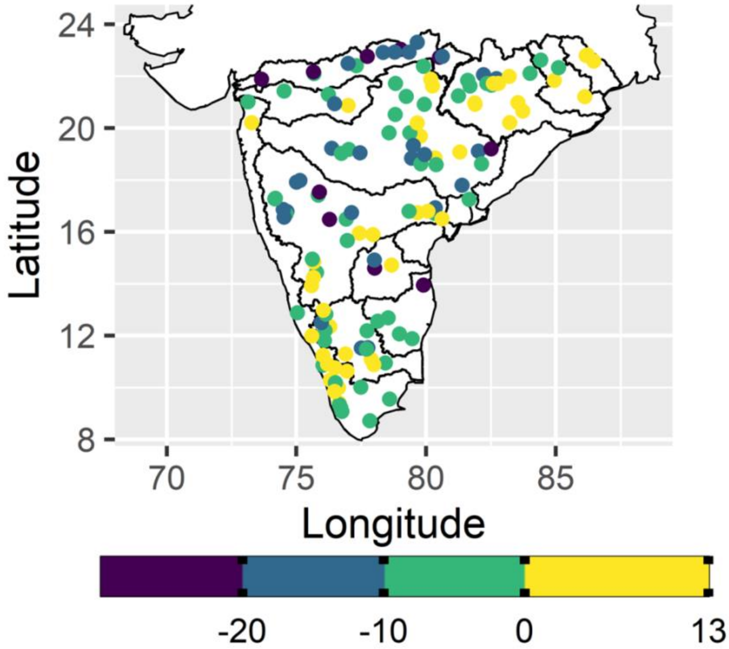

Figure S5. Trends in annual flood magnitudes at 137 streamflow gauges in Peninsular India.

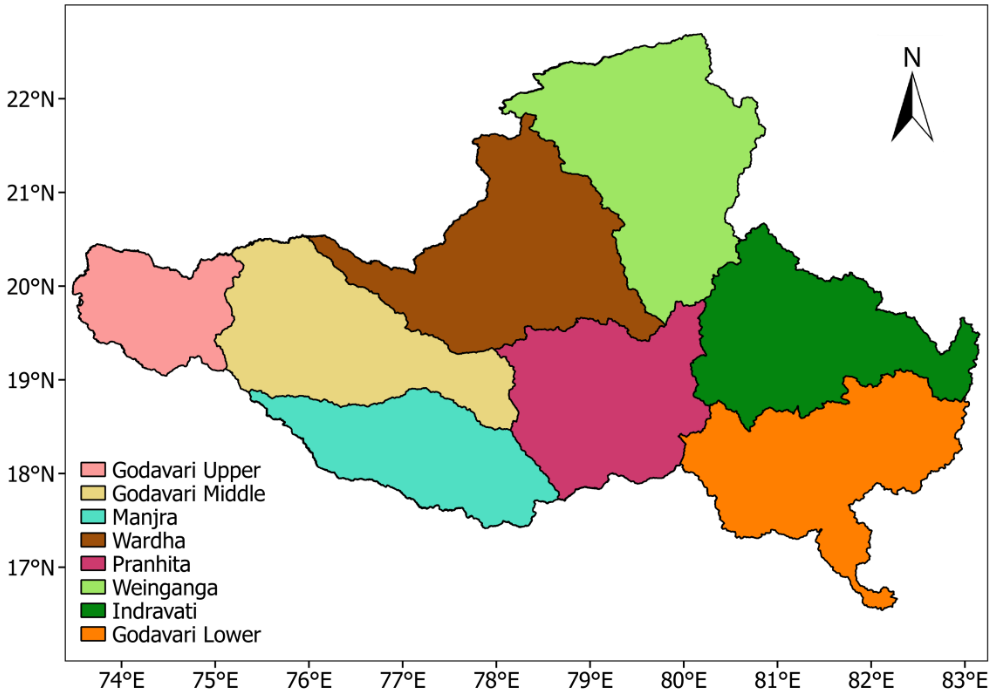

Figure S6. Subbasins of Godavari river basin of Peninsular India.

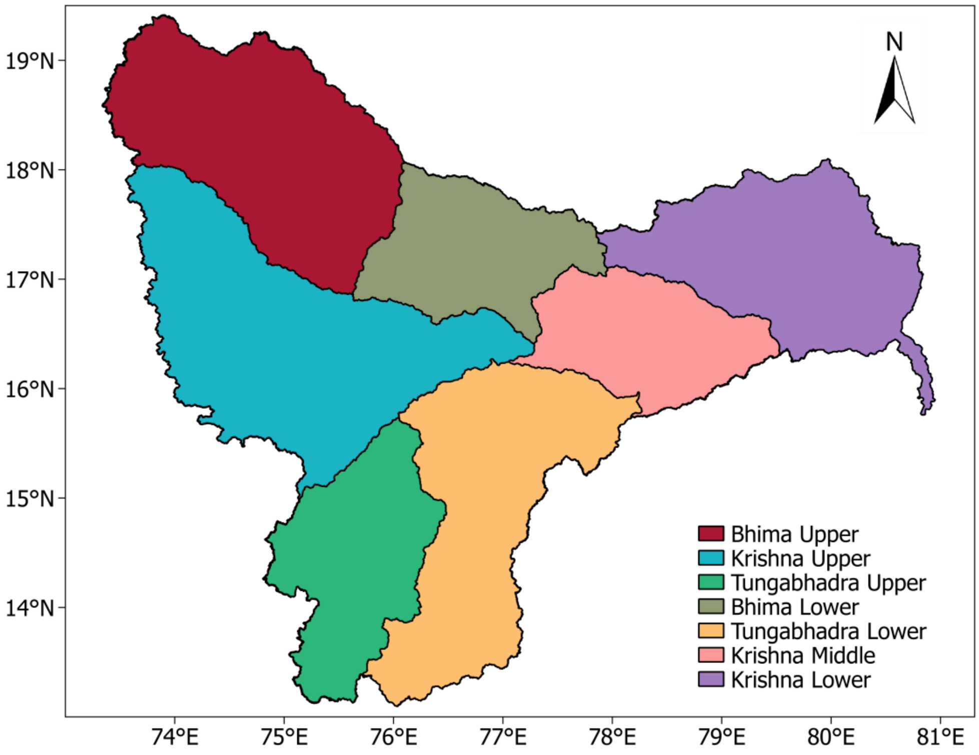

Figure S7. Subbasins of Krishna river basin of Peninsular India.
